# Prevalence of Chronic Hepatitis B and C Infection in Mongolian Immigrants in the Washington, District of Columbia, Metropolitan Area, 2016–2017

**DOI:** 10.5888/pcd16.180104

**Published:** 2019-01-24

**Authors:** Emmeline Ha, Frederic Kim, Janice Blanchard, Hee-Soon Juon

**Affiliations:** 1 School of Medicine and Health Sciences, The George Washington University, Washington, District of Columbia; 2 Hepatitis B Initiative of Washington DC, Washington, District of Columbia; 3 Department of Medical Oncology, Thomas Jefferson University, Philadelphia, Pennsylvania

## Abstract

**Introduction:**

Mongolia has the highest liver cancer incidence in the world. Hepatocellular carcinoma is the most prevalent primary liver cancer, and the most common risk factors are hepatitis B virus (HBV) or hepatitis C virus (HCV) infection. Although viral hepatitis occurs mostly in the developing world, migration of people from high prevalence countries contributes to the health outcomes of the United States. Data on Mongolian Americans is limited. The objective of this study was to estimate HBV and HCV infection prevalence among Mongolia-born immigrants living in the Washington, District of Columbia, metropolitan area.

**Methods:**

We tested Mongolia-born immigrants for chronic hepatitis at community-based screening events from 2016 to 2017. Descriptive statistics were generated to describe the screening results. Bivariate analysis was conducted to examine the relationship between hepatitis prevalence and sociodemographic characteristics.

**Results:**

Of 634 participants, most did not speak English primarily, were uninsured, and did not have a regular primary care provider. Eighty-two participants (12.9%) had chronic HBV or HCV infection after accounting for HBV and HCV co-infection. Thirty-nine (6.2%) were chronically infected with HBV, and 233 (36.8%) were susceptible to HBV. Sixty-three (9.9%) participants were positive for HCV exposure, and 45 (7.1%) had confirmed chronic HCV infection. While no sociodemographic characteristics were associated with HBV infection, age and primary spoken language (Mongolian) were significantly associated with HCV exposure.

**Conclusion:**

Foreign-born immigrants such as Mongolian Americans have a high prevalence of chronic viral hepatitis infection. Targeted screening, vaccination, and treatment programs can help decrease immigrant risk for developing hepatocellular carcinoma.

**Summary**

**What is already known on this topic?**

Mongolia has the highest liver cancer incidence in the world. The most common risk factor for hepatocellular carcinoma is chronic viral hepatitis infection. 

**What is added by this report?**

Using data from community-based screenings, we are the first to report the prevalence of chronic hepatitis B and hepatitis C infection in Mongolian American immigrants, an understudied minority population.

**What are the implications for public health practice?**

Foreign-born immigrants have a high prevalence of chronic viral hepatitis infection. Targeted screening, vaccination, and treatment programs can help decrease immigrant risk for developing hepatocellular carcinoma. 

## Introduction

Liver cancer is the second most common cause of death from cancer worldwide (1). Hepatocellular carcinoma (HCC) is the most prevalent primary liver cancer, and its incidence is increasing (2). Infection from hepatitis B virus (HBV) or hepatitis C virus (HCV) is the greatest risk factor for developing HCC (3,4). Mongolia has the highest liver cancer incidence in the world at a rate of 78.1 cases per 100,000 people (5). In comparison, the liver cancer incidence for the United States is 6.1 per 100,000 (5). Furthermore, in Mongolia, 99.3% of all cancer cases including liver cancer are attributed to infection (6).

In 2017, a nationwide survey in Mongolia found that 19.4% of the adult population was infected with either HBV or HCV (7). Prevalence of HBV infection in Mongolia is approximately 9% to 11.8% (7–9) and HCV prevalence is approximately 8.5% to 11.0% (7,9). In contrast, HBV infection prevalence in the United States is estimated at 0.3% to 0.7% and HCV prevalence at 1% to 1.6% (1,10). These differences are due to higher rates of perinatal and iatrogenic transmission in developing countries like Mongolia (9–11).

While most viral hepatitis cases occur in the developing world, migration of people from high prevalence countries contributes to the public health system of their host countries (10). For instance, three-quarters of HBV infections in the United States are among foreign-born persons (12). Studies have also found that Asian Americans and Pacific Islanders have a higher incidence of HCC compared with other groups in the United States (13,14).

Chronic viral hepatitis infection is preventable and treatable. The objective of this study was to estimate HBV and HCV infection prevalence among Mongolia-born immigrants in the Washington, District of Columbia (DC), metropolitan area and to identify sociodemographic factors associated with infection. Findings from this study may support initiatives to prevent and treat viral hepatitis in migrant populations and ultimately decrease HCC incidence.

## Methods

### Data collection

We analyzed retrospective data obtained from community health screenings held from 2016 through 2017 for Mongolian immigrants living in the Washington metropolitan area (Maryland, Virginia, and Washington, DC). These events were partnered with local Mongolian community leaders to help gather participation. They were held at a community health center and were promoted as free health screenings that included hepatitis B and C status, lipid profile levels, and point-of-care blood glucose checks. Advertisements that were culturally tailored and linguistically sensitive were distributed among religious organizations, businesses, media outlets, and community centers.

On the day of the events, screening staff included community partner representatives, bilingual volunteers, and trained phlebotomists. Health educators were also present to provide further information about chronic hepatitis infection and the importance of screening. Event participants aged 18 years or older gave consent and were given a brief survey about their demographic information, which included questions about country of birth, age, sex, year of arrival to the United States, health insurance status, and established access to a regular primary care provider. These surveys were offered in both English and Mongolian. Free on-site HBV and HCV testing was then administered by the phlebotomists. Participants were contacted within 2 to 3 weeks with their results. Study participants who tested positive for chronic hepatitis were contacted by a care coordinator to provide medical follow-up. In addition, participants who did not have immunity against HBV were referred to programs that provided the complete vaccination series free of charge. This study was approved by The George Washington University Institutional Review Board.

### Measures

Serology results included status of hepatitis B surface antigen (HBsAg), hepatitis B surface antibody (anti-HBs), and hepatitis C antibody (anti-HCV). Participants who were positive for anti-HCV also had results for reflex HCV RNA quantitative testing through reverse transcription polymerase chain reaction assay. The sensitivity threshold for HCV RNA was at 15 IU/mL.

For HBV screening, participant results were grouped as follows: 1) HBV infected (positive HBsAg), 2) susceptible to HBV infection (negative HBsAg and negative anti-HBs), and 3) immune to HBV infection (negative HBsAg and positive anti-HBs). For HCV screening, participant results were grouped by 1) HCV infected (positive anti-HCV with reflex quantitative HCV RNA ≥15 IU/mL), 2) resolved HCV infection (positive anti-HCV with reflex quantitative HCV RNA <15 IU/mL), and 3) negative (negative anti-HCV).

### Analysis

Descriptive statistics were generated to describe the screening results. Bivariate analysis was then conducted to examine the relationship of prevalence and categorical demographic characteristics using χ^2^ tests and Fisher exact tests. All statistical analyses were performed with Stata, version 14 (StataCorp LLC). Significance was set at *P* < .05.

## Results

A total of 637 immigrants were screened. Three were excluded from further analysis because birth country was outside of Mongolia. The final study sample size was 634. Of the study participants, 39 (6.2%) were chronically infected with HBV, 233 (36.8%) were susceptible to infection, and 362 (57.1%) were immune to infection (Table 1). Sixty-three (9.9%) participants tested positive for anti-HCV, indicating previous exposure to HCV. Among these 63 seropositive for anti-HCV, 45 (71.4%) had detectable HCV-RNA and 18 (28.6%) did not. The total number of participants with chronic HCV infection was thus 45 (7.1%). Five hundred and seventy-one (90.0%) were negative for previous HCV exposure. Two participants (0.3%) were positive for both HBV and HCV infection.

**Table 1:** Hepatitis Results for Mongolia-born Screening Participants (N = 634) in the Washington, District of Columbia, Metropolitan Area, 2016–2017

Screening Result	n	% (95% Confidence Interval)
**Hepatitis B**		
Chronic infection (HBsAg positive)	39	6.2 (4.5–8.4)
Susceptible to infection (HBsAg negative and anti-HBs negative)	233	36.8 (32.7–40.9)
Immune (HBsAg negative, anti-HBs positive)	362	57.1 (52.7–61.4)
**Hepatitis C**		
Exposed (anti-HCV positive and HCV RNA <15 IU/mL)	63	9.9 (7.7–12.8)
Chronic infection (anti-HCV positive and HCV RNA ≥15 IU/mL)	45	7.1 (5.6–8.9)
Negative (anti-HCV negative)	571	90.0 (87.9–91.8)

Abbreviations: anti-HBs, hepatitis B surface antibody; anti-HCV, hepatitis C antibody; HBsAg, hepatitis B surface antigen; HCV, hepatitis C virus.

The mean (standard deviation) age of participants was 41 (11.4) years. Most participants were women (n = 366, 57.7%) (Table 2). About half of the participants (n = 318, 50.2%) were living in the United States for 5 years or less, and the largest group was those in the United States for 1 to 5 years (n = 253, 39.9%). Most of the sample group spoke Mongolian primarily (n = 434, 68.4%), while 200 (31.6%) reported English as their primary language. Most were uninsured (n = 564, 89.0%) and did not have an established regular primary care provider (n = 569, 89.8%).

**Table 2:** Demographic Characteristics of Mongolia-born Screening Participants (N = 634) in the Washington, District of Columbia, Metropolitan Area, 2016–2017

Characteristic	Total Participants, N (%)	Hepatitis B Infected^a^		Hepatitis C Exposed^b^	
		n (%)	*P* Value	n (%)	*P* Value
**Age, y**					
18–30	125 (19.7)	8 (6.4)	.44^c^	4 (3.2)	<.001^c^
31–40	183 (28.9)	10 (5.5)		8 (4.4)	
41–50	198 (31.2)	9 (4.6)		27 (13.6)	
51–60	93 (14.7)	9 (9.7)		14 (15.1)	
≥61	35 (5.5)	3 (8.6)		10 (28.6)	
**Sex**					
Female	366 (57.7)	25 (6.8)	.41^c^	40 (10.9)	.33^c^
Male	268 (42.3)	14 (5.2)		23 (8.6)	
**Years in the United States^d^**					
<1	65 (10.3)	6 (9.2)	.64^c^	9 (13.9)	.26^c^
1–5	253 (39.9)	18 (7.1)		24 (9.5)	
6–10	104 (16.4)	6 (5.8)		5 (4.8)	
11–14	136 (21.5)	6 (4.4)		17 (12.5)	
≥15	68 (10.7)	3 (4.4)		7 (10.3)	
**Primary spoken language**					
English	200 (31.6)	8 (4.0)	.13^c^	10 (5.0)	.011^c^
Mongolian	434 (68.4)	31 (7.1)		53 (12.2)	
**Health insured**					
Yes	70 (11.0)	1 (1.4)	.11^e^	7 (10.0)	.98^c^
No	564 (89.0)	38 (6.7)		56 (9.9)	
**Regular primary care provider**					
Yes	65 (10.2)	2 (3.1)	.41^e^	8 (12.3)	.50^c^
No	569 (89.8)	37 (6.5)		55 (9.7)	

a Hepatitis B surface antigen positive.

b Hepatitis C antibody positive.

c χ^2^ analysis.

d Eight participants had missing data for years living in the United States, including 1 participant who was positive for hepatitis C exposure.

e Fisher exact test analysis.

When stratifying for sociodemographic factors, no characteristics were significantly associated with HBV infection (Table 2). However, age and primary spoken language were significantly associated with HCV exposure (*P* < .05). The highest prevalence of HCV exposure was among those aged 51 to 60 years (14 positive of 93; 15.1%) and those aged 61 years or older (10 positive of 35; 28.6%). Prevalence was lower among those aged 18 to 30 years (4 positive of 125; 3.2%) and those aged 31 to 40 years (8 positive of 183; 4.4%). Of the 434 participants who reported Mongolian as their primary spoken language, 53 (12.2%) tested positive for HCV exposure. For the 200 who reported English as their primary spoken language, 10 (5.0%) tested positive.

## Discussion

This study is the first to report viral hepatitis prevalence for both HBV and HCV in Mongolia-born individuals living in the United States. Of the 634 immigrants screened, 12.9% were found to have chronic HBV and/or HCV infection after accounting for HBV/HCV co-infection (n = 2). This is lower than the endemic prevalence of 19% in Mongolia but higher than that of the general United States population (7). These findings support other studies that have shown foreign-born populations uniquely contribute to chronic hepatitis infection prevalence in the United States (14,15).

The prevalence of HBV in Asian Americans has been extensively studied through surveys, community-based participatory initiatives, and outreach screenings (16–18). Approximately 7% of Asian Americans have chronic HBV (15,17). However, limited data exist for those of Mongolian ethnicity. Of those screened in this study, 6.2% were positive for chronic HBV infection. Only one other study reports HBV prevalence in Mongolian Americans, where 13 (6.8%) of 190 participants in Alameda County, California, were HBsAg positive (16). These rates for Mongolian immigrants are lower than those reported by a 2012 systematic review of chronic HBV infection in foreign-born populations of the United States (15). That study estimated distinct prevalence rates for immigrant communities from 102 different countries, and Mongolians were grouped as other nonspecified Eastern Asian at a prevalence of 8.97% (15). The discrepancies in HBV infection prevalence reveal the nuances of current data on viral hepatitis epidemiology. The true burden of chronic HBV in the United States is limited by inadequate representation of foreign-born populations that face barriers to care including health literacy and access (10,15). The Centers for Disease Control and Prevention (CDC) and US Preventive Services Task Force (USPTF) recommend HBV testing for persons born in countries where HBV infection is endemic at prevalence of 2% or higher (12,15).

Over one-third (36.8%) of participants had no immunity to HBV. Universal HBV immunization for newborns was not implemented in Mongolia until 1991 (19). This may have contributed to the lack of immunity for this study’s immigrant group of predominantly middle-aged adults (mean age >40 y). Since HBV is a strong risk factor for the development of HCC, vaccination can prevent liver cancer (11,14). While vaccination initiatives substantially decreased the incidence of HBV in children worldwide, unvaccinated adults still represent an ongoing risk for HBV infection and HCC (14,19).

More than half (57.1%) of the study participants were immune to HBV infection, identified by positive anti-HBs in serology. Because hepatitis B core antibody (anti-HBc) was not measured, it remains unclear if these individuals were immune secondary to vaccination history or recovery from previous infection. This finding was varied compared with other studies of Asian American immigrants, whose prevalence of HBV immunity was between 46% and 64% (16,18,20). The most recent published data in Mongolia estimates 37% prevalence of positive anti-HBs for adults aged 20 years or older (7).

This study is the first to report HCV prevalence in Mongolia-born individuals in the United States. Of the Mongolian immigrants screened in this study, 7.1% were positive for chronic HCV infection. HCV in Asian Americans has been understudied secondary to overshadowing disparities in HBV prevalence (21,22). An estimated 3% to 6% of the Asian American population is seropositive for anti-HCV (20,23). CDC and USPTF recommend HCV screening for those in the 1945 through 1965 birth cohort and those with high-risk factors including history of past or current injection drug use, blood transfusion before 1992, long-term hemodialysis, HIV infection, maternal HCV infection, or occupational exposure (22,23). However, multiple studies have found that these traditional risk factors for HCV are not evident in seropositive Asian Americans and thus they are often underdiagnosed for HCV (22,23). HCV prevalence in Mongolia has been associated with iatrogenic factors including medical equipment sterilization and disinfection (7,9). Single-use syringes were not nationally mandated in health care facilities until 1995 (9). Mongolia did not screen for HCV in transfusions until 1997 (24). This study’s high prevalence of HCV in Mongolia-born immigrants suggests that factors unique to county-of-birth contribute to risk for viral hepatitis infection. Screening guidelines for HCV should be expanded to include foreign-born persons whose native country’s HCV prevalence is 2% or higher (21,22).

Demographic characteristics of the study participants indicated disparities common for immigrant populations in the United States. Most participants had a primary spoken language other than English, were uninsured, and did not have an established regular primary care provider. This study used data from community-based screening initiatives that were able to target a hard-to-reach population such as Mongolia-born immigrants. Community-based efforts thus provide opportunities for engaging high-risk, foreign-born individuals who are limited by cultural, economic, and environmental barriers (15).

While no sociodemographic characteristics were associated with HBV infection, age and primary spoken language were significantly associated with HCV exposure. Older age was associated with HCV, reflecting similar age distributions for studies in Mongolia (7–9). This is consistent with the issues of iatrogenic transmission in Mongolia. Those who are older are more likely than younger people are to have been exposed to unsterile medical equipment or blood products before the implementation of regulations. Participants who reported Mongolian as their primary spoken language were also more often associated with HCV exposure than those who reported English as their primary spoken language. This suggests the role of acculturation in infection risk, where those who speak English may be better able to navigate health services and have increased health literacy.

There are several limitations in this study. First, chronic hepatitis prevalence was only assessed in Mongolian immigrants who were living in the Washington metropolitan area. In 2015, the population of Mongolian Americans was approximately 21,000, with Chicago and Washington, DC, as the top metropolitan areas with approximately 3,000 Mongolian Americans each (25). Viral hepatitis infection prevalence in Mongolia-born immigrants may vary depending on settled area. In addition, the study sample may have selection bias secondary to data obtained from community-based screenings, which recruited voluntary participants by targeted local advertising. This nonprobability sampling method may not represent the entire Mongolia-born immigrant population of the Washington metropolitan area. Furthermore, HBV-positive or HCV-positive individuals who were aware of their viral hepatitis status may have been less likely to participate in screening events, and this could have resulted in underestimation of prevalence. The use of screening data from Mongolian community-based initiatives also did not allow for screening the general population of the area. This resulted in the lack of a comparison group for the study. Moreover, chronic hepatitis infection surveillance is not mandated, and reporting varies nationwide (12). No published data from the Washington metropolitan area were available to provide adequate comparison of prevalence. Lastly, the prevalence rates determined do not consider severity of hepatitis infection as this was out of scope for the study. Thus, liver sequelae including HCC in the participants found to be HBV or HCV seropositive was not addressed.

The strength of this study was determining the prevalence of both HBV and HCV in an understudied population of immigrants, whose native country has endemic viral hepatitis and the highest incidence of HCC worldwide. Through community-based initiatives, over 600 immigrants were screened for chronic hepatitis B and C in this study. Most participants of this study did not speak English primarily and had health barriers including lacking insurance or a primary health care provider. These findings support the need for targeted screening, vaccination, and treatment programs for foreign-born individuals such as Mongolian immigrants, among whom prevalence is high compared with that of the general US population. Furthermore, the high HCV infection prevalence in immigrants reflects the infection risks of their county of birth, and this should be addressed in the screening guidelines. Overall, the high prevalence of viral hepatitis in immigrant populations ultimately contributes to the hepatitis disease burden in the United States.
